# Evidence of multiple mechanisms providing carbamate and organophosphate resistance in field *An. gambiae* population from Atacora in Benin

**DOI:** 10.1186/s13071-014-0568-5

**Published:** 2014-12-02

**Authors:** Rock Aïkpon, Michel Sèzonlin, Razaki Ossè, Martin Akogbéto

**Affiliations:** Centre de Recherche Entomologique de Cotonou (CREC), 06 BP 2604, Cotonou, Bénin; Faculté des Sciences et Techniques, Université d’Abomey Calavi, Calavi, Bénin

**Keywords:** Resistance mechanisms, Carbamate, Organophosphate, *Anopheles gambiae*, Benin

## Abstract

**Background:**

Insecticide resistance in *Anopheles gambiae* s.l is a major concern to malaria vector control programmes. In West Africa, resistance is mainly due to target–site insensitivity arising from a single point mutation. Metabolic-based resistance mechanisms have also been implicated and are currently being investigated in west Africa. The aim of this study is to better understand the origins of carbamate and organophosphate resistance in *An. gambiae* population from Atacora, Benin in West Africa.

**Methods:**

Anopheles mosquitoes were reared from larvae collected in two districts (Kouandé and Tanguiéta) of the Atacora department. Mosquitoes were then exposed to WHO impregnated papers. Four impregnated papers were used: carbamates (0.1% bendiocarb, 0.1% propoxur) and organophosphates (0.25% pirimiphos methyl, 1% fenitrothion). PCR assays were run to determine the members of the *An. gambiae* complex, as well as phenotypes for insensitive acetylcholinesterase (AChE1). Biochemical assays were also carried out to detect any increase in the activity of enzyme typically involved in insecticide metabolism (oxidase, esterase and glutathion-S-transferase).

**Results:**

769 female of *An. gambiae* mosquitoes from Kouandé and Tanguiéta were exposed to bendiocarb, propoxur, pirimiphos methyl and fenitrothion. Bioassays showed resistance with low mortality to bendiocarb (78.57% to 80.17%), propoxur (77.21% to 89.77%), and fenitrothion (89.74% to 92.02%). On the other hand, the same populations of *An. gambiae* from Kouandé and Tanguiéta showed high susceptibility to pirimiphos methyl with recorded mortality of 99.02% and 100% respectively. The low rate of ace-1R allele frequency (3.75% among survivors and 0.48% among dead) added to the high proportion of homozygous susceptible specimens which survived the WHO bioassays (8/28), suggest that the ace-1 mutation could not entirely explain *Anopheles gambiae* resistance to carbamate and organophosphate. Biochemical assays suggest that resistance in this population is mediated by metabolic resistance with elevated level of GST, MFO and NSE compared to a susceptible strain *An. gambiae* Kisumu.

**Conclusions:**

*Anopheles gambiae* populations resistance from Atacora is multifactorial and includes target-site mutation and metabolic mechanism. The co-implication of both resistance mechanisms in *An. gambiae* s.l may be a serious obstacle for the future success of malaria control operations based on LLINs and IRS.

## Background

Malaria is a main cause of mortality and morbidity and *Anopheles gambiae* is one of the major vectors of this desease in sub-Saharan Africa [1]. The current effective vector control tools include the use of Long Lasting Insecticide Nets (LLIN) and Indoor Residual Spraying (IRS) [2]. The success of such interventions requires a good knowledge of vector populations particulary their susceptibility status to the main insecticides used for such control programmes in order to detect and monitor resistance to these insecticides.

Pyrethroids are the only group of insecticides currently recommended for net treatment and the other classes of insecticides (organochlorine, carbamate and organophosphate) are applied for IRS [3,4]. The main problem with LLINs and IRS is the development of insecticide resistance, particularly pyrethroid-resistance by several populations of *An. gambiae* [5-8]. Recently, the emergence of resistance in populations of *An. gambiae* to common classes of insecticides used in public health has been reported in many countries in Africa, including Côte d’Ivoire [5,9], Kenya [10], Benin [11,12], Niger [13], Burkina Faso [14], Mali [15], Nigeria [16,17], South Africa [18] and Cameroon [19]. More recently in Benin, several studies pointed out *An. gambiae* resistance to bendiocarb [20,21] and showed that the mean *Ace-1* mutation frequency had increased significantly from 2010 to 2012 after two years of IRS campaign using bendiocarb in Atacora region in the northern part of the country [22,23]. However, these studies cleary showed the evidence of bendiocarb resistance in *An. gambiae* populations but mentioned the *ace-1R* mutation could not entirely explain the resistance to bendiocarb observed.

The aim of this study is to better understand the origins of carbamate and organophosphate resistance in *An. gambiae* population from Atacora. We conducted bioassays, molecular and biochemical analysis to update resistance status in *An. gambiae* and detect other potential mechanisms of resistance that might have evolved.

## Methods

### Study area

**Figure 1:**
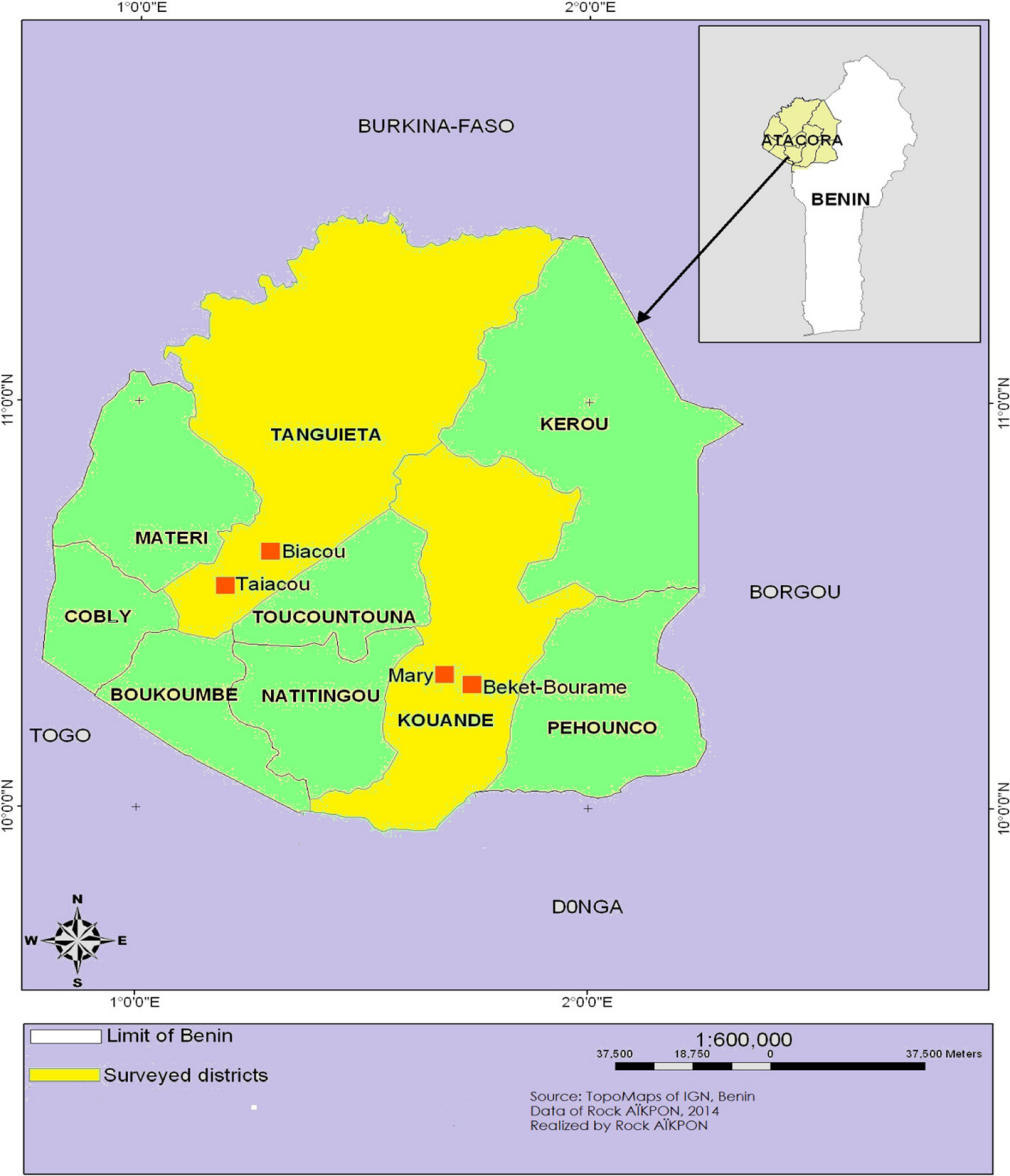
**Map of the departement of Atacora showing the localities where Anopheline mosquitoes were collected.**

The study was carried out in Atacora region located in the North-west of Benin and includes two districts: Kouandé and Tanguiéta (Figure 1). The two districts covered 5,128 km2 and had an estimated population of 198,956 in 2012. Atacora region has a sub-equatorial type climate with one dry season (December-May) and only one rainy season (June to November). The annual mean rainfall is 1,300 mm and the mean monthly temperature ranges between 22 and 33°C. The region is irrigated by three major rivers: the Mekrou, the Pendjari and the Alibori. The major economic activity is agriculture and it is characterized by the production of cotton and millet where various classes of pesticides are used for pest control. Since 2011 onwards the department has conducted a large scale Indoor Residual Spraying (IRS) campaign.

### Mosquito collections

*Anopheles gambiae s.l.* larvae were collected in 2 districts and in each district, two villages were selected randomly. At each locality chosen, Anopheline larvae were collected from various natural breeding sites including ground pools, gutters, puddles and abandoned potholes, at the begining of the rainy season during June 2014. Water was scooped using a plastic scoop and poured into small transparent plastic bowls. A strainer was used to sieve and pool together the third and fourth instar larvae in order to have sufficient adult emergence of the same physiological age. The mosquito larvae collected were transported in well labelled plastic bottles to the laboratory of the Centre de Recherche Entomologique de Cotonou, Benin (CREC) where they were maintained at 28 ± 2 C and 72 ± 5% relative humidity. A laboratory susceptible strain of *An. gambiae* Kisumu was used as a reference strain to compare the susceptibility levels of the field populations.

### Insecticide susceptibility tests

Mosquitoes collected were assayed using WHO discriminating dosages with four insecticides of technical grade quality: two carbamates (0.1% propoxur, 0.1% bendiocarb) and two organophosphates (1% fenitrothion, 0.25% pirimiphos méthyl). Four batches of 25 unfed females, aged 2–5 days, were exposed to the diagnostic doses of insecticide treated papers for 60 min at 27 ± 1°C and 80% relative humidity. The twenty-five females of *An. gambiae* were introduced into each tube and monitored at different time intervals (10, 15, 20, 30, 45, 60 minutes), the number “knocked-down” were recorded. After one hour exposure, mosquitoes were transferred into holding tubes and provided with cotton wool soaked with a 10% honey solution. Batches exposed to untreated papers were used as control. Mortalities were recorded after 24 hours and the susceptibility status of the population was graded according to the WHO protocol [24]. Dead and surviving mosquitoes from this bioassay were kept separately in eppendorf tubes containing silica gel and stored at −20°C for further molecular analysis.

### Species identification and PCR detection of *Ace-1R* mutation

Live and dead specimens of *An. gambiae* from the bioassay tests were subjected to the *An. gambiae* species specific PCR assays for species identification [25,26]. The PCR-RFLP diagnostic test was used to detect the presence of G119S mutation (ace.1R gene). Mosquito genomic DNA was amplified using the primers Ex3AGdir 5’ GATCGTGGACACCGTGTTCG3’ and Ex3AGrev 5‘AGGATGGCCCGCTGGAACAG3’ according to [27]. One microlitre of total DNA extracted from a single mosquito was used as a template in a 25 ml PCR reaction containing Taq DNA polymerase buffer, 0.2 mM dNTP and 10 pmol of each primer. The PCR conditions were 94°C for 5 min and then 35 cycles of (94°C for 30 s, 54°C for 30 s and 72°C for 30 s) with a final 5 min extension at 72°C. Fifteen microlitres of PCR product were digested with 5U of AluI restriction enzyme (Promega) in a final volume of 25 ml. The PCR fragments were fractionated on a 2% agarose gel stained with ethidium bromide and visualized under UV light.

### Biochemical analysis

Biochemical assays were performed to compare the levels of activity of mixed function oxidases (MFO), non-specific esterases (NSE) using a-naphtyl acetate as a substrate and glutathione S-transferases (GST) [28] in the *An. gambiae s.s*. susceptible Kisumu and the field population from Tanguiéta and Kouandé districts. Mosquitoes used for the biochemical analysis have not been exposed to any insecticides prior to the assay.

Detoxifying enzyme activities were measured on single mosquitoes (N = 50) from each test locality, which were stored at 280uC within 24 h from emergence (above). Each mosquito was ground on ice in 200 μl of distilled water and the homogenate was centrifuged at 1,4000 rpm for 2 mins. Two 10 ml replicates of supernatant were transferred into two adjacent wells of a microtitre plate for non-specific esterases (NSEs), gluthatione Stransferases (GSTs). Monooxygenases assays were performed with two 20 ml replicates of supernatant.

#### Non-specific esterases (NSEs)

Non-specific esterase activity was measured using a-naphtol acetate (aNa) and b-naphtol acetate (bNa). In each replicate well, 90 ml of phosphate buffer (PBS, pH = 6.5) and 100 ml of 0.6 M aNa (or bNa) were added to 10 ml of centrifuged mosquito homogenate. After 30 min incubation, 100 ml of Fast Garnett BC solution (8 g Fast Garnett Salt +10 ml distilled water) was added to stop the reaction. The concentration of the final product was determined at 550 nm as an endpoint calculated from standard curves of a- and b-Naphtol, respectively.

#### Glutathione-S-transferases (GST)

To measure glutathione- S-transferase (GST) activity in mosquitoes, 200 ml of GSH/CDNB working solution (100 ml of an extemporaneous solution of 0.6% weight/volume reduced glutathione in 0.1 M sodium phosphate buffer pH = 6.5 + 0.013 g of 1-chloro-2, 4-dinitrobenzene diluted in 1 ml of 70% methanol) were added to each replicate of mosquito homogenate. The reaction was read at 340 nm immediately as a kinetic assay for 5 minutes. An extinction coefficient of 5.76 mM^−1^ (corrected for a path length of 0.6 cm) was used to convert absorbance values to moles of product. GST specific activity was reported as the rate of formation of GSH produced in mmol.min^−1^.mg^−1^ protein.

#### Oxydases (Cytochrome P450)

Cytochrome P450 activity was determined using the heme-peroxidase assay according to Brogdon et al. [29]. The assay detects the elevation in the amount of heme, which is then converted into equivalent units of cytochrome P450. Eighty ml of 0.625 M potassium phosphate buffer (pH = 7.2) were added to 20 ml of mosquito homogenate together with 200 ml Tetramethyl Benzidine solution (0.011 g 3,3’,5,5’ Tetramethyl Benzidine in 5 ml of 70% methanol +15 ml sodium acetate buffer 0.25 M pH = 5.0); 25 ml of 3% hydrogen peroxide were then added and the mixture was incubated for 30 min at room temperature. Absorbance was read at 630 nm and values calculated from a standard curve of cytochrome C.

### Data analysis

The resistant status of mosquito samples was determined according to the WHO criteria [23]:

- Mortality rate is > 98%: the population was considered fully susceptible
- Mortality rates ranged between 90 - 98%: resistance suspected in the population
- Mortality rates < 90%, the population was considered resistant to the tested insecticides

Allelic frequencies of G119S mutation were analysed using the version 1.2 of Genepop [30].

To assess if the mutation frequencies were identical across populations, the test of genotypic differentiation was performed [31]. Biochemical assay data (enzymatic activity per mg of protein) of wild specimens of An. gambiae s.l were compared to the Kisumu susceptible strain using Mann–Whitney non-parametric *U-*test (Statistica software).

## Results

### Bendiocarb and organophosphate susceptibility status of *Anopheles gambiae s.l*

**Figure 2:**
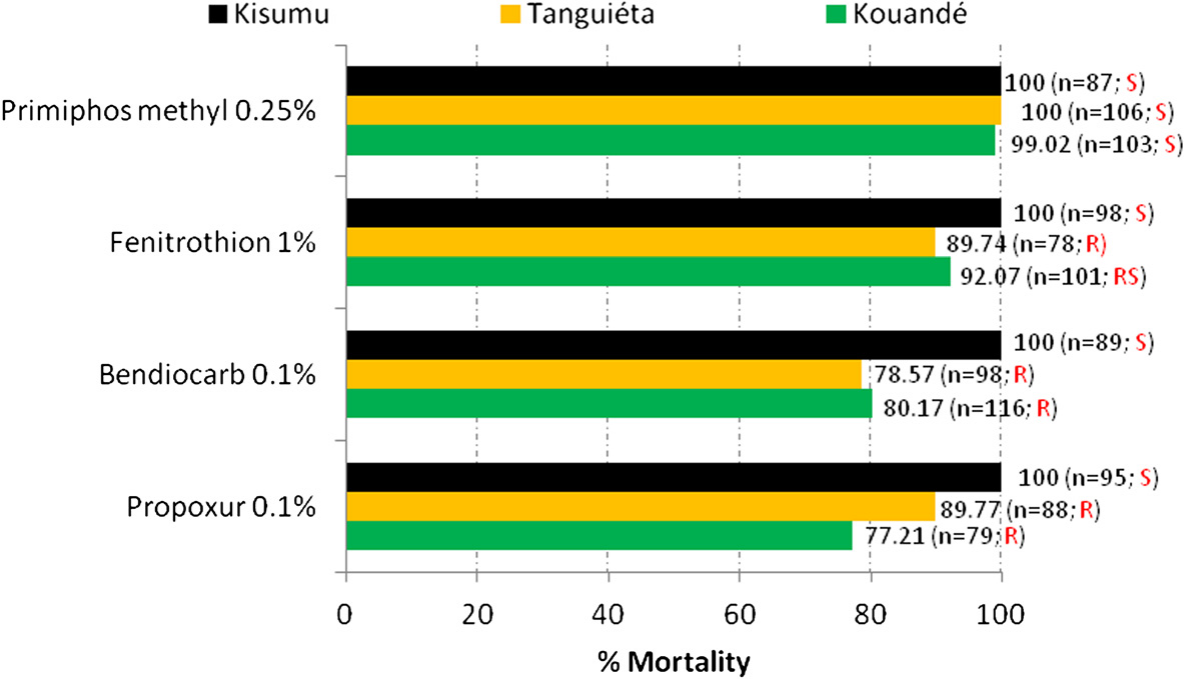
**Mortality rates in** ***Anopheles gambiae*** **24-hours post exposure to propxur 0.1%, bendiocarb 0.1%, fenitrothion 1% and pirimiphos methyl 0.25%.** S = susceptible; R = resistant; RS = resitance suspected.

A total of 769 female of *An. gambiae* mosquitoes (3–5 days old) from Kouandé and Tanguiéta were exposed to bendiocarb (n = 214), propoxur (n = 167), pirimiphos methyl (n = 209) and fenitrothion (n = 179). Mortality rates of the Kisumu reference strain to all insecticides was 100% (Figure 2). In contrast, wild mosquitoes were high resistant with mortality rates less than 90% for bendiocarb (80.17% and 78.57%) and propoxur (77.21% and 89.77%) in Kouandé and Tanguiéta respectively (Figure 2). As for the organophosphates, susceptibility to pirimiphos-methyl was assessed on all populations. Mortality rate were higher than 98% as per WHO criteria with 99.02% recorded in Kouandé and 100% in Tanguiéta. However, fenitrothion resistance was detected in Kouandé and Tanguiéta, with mortality rates of 92.07% and 89.74% respectively.

### PCR- species identification

One hundred mosquitoes were identidied to *Anopheles gambiae* species. All PCR analysis identifying *An. gambiae s.l* species in this study confirmed that all mosquitoes belonging to *An. gambiae s.l* were *An. gambiae.*

### Involvement of ace-1R mutation in providing carbamate and organophosphate resistance

**Figure 3:**
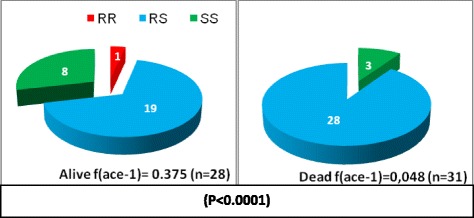
**Ace-1 genotypes distribution among live and dead** ***An. gambiae s.s*** **individuals from WHO susceptibility test to carbamate and organophosphate.**

To assess the role of the ace-1R allele in conferring carbamate and organophosphate resistance in *An. gambiae* population, the ace-1R genotype was determined for dead and alive mosquitoes detected in the WHO bioassay using carbamate and organophosphate (Figure 3). A total of 59 mosquitoes were tested of which 31 dead and 28 alive. Among both bioassay survivors and non-survivors, all ace-1R genotypes (RR, RS and SS) were found. However, the homozygous resistant genotype RR was only found among the bioassay survivors, and the heterozygote genotype RS was the most prevalent genotype among the bioassay survivors. Although there was a significant ace-1R genotype differentiation between bioassay survivors and non-survivors (p < 0.0001), homozygous susceptible mosquitoes were found among bioassay survivors.

### Biochemical assays

**Figure 4:**
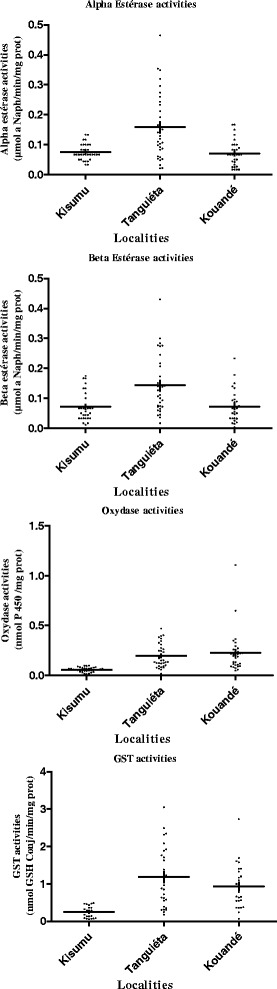
**Detoxifying enzyme activities in** ***An. gambiae*** **mosquitoes collected form two districts in Ataccora departement.**

Biochemical assays were successfully performed on the two *An. gambiae* populations (Tanguiéta and Kouandé) from Atacora. Figure 4 shows the mean level of enzymatic activity (MFO, NSE and GST) in field-collected mosquitoes compared to the reference susceptible strain Kisumu. All sampled populations showed a significantly higher MFOs activity (expressed in cytochrome P450 units) than the susceptible reference strain Kisumu (p < 0.005). The level of esterase activity (using alpha and beta naphtyl acetate as a substrate) did not reveal any significant differences between Kisumu and Kouandé *An. gambiae* population (p = 0.65). Overall, the mean level of alpha and beta esterase activity in Tanguiéta was significantly higher than that of the susceptible strain (p < 0.05). Besides, all sampled populations showed a significantly higher GST activity than the susceptible reference strain Kisumu (p < 0.05).

## Discussion

This study highlighted the diversity of carbamate and organophosphate resistance in *Anopheles gambiae s.l*. populations from Atacora in Benin. The results presented here show that carbamate and organohosphate resistance in sampled *Anopheles gambiae* populations is multifactorial and includes target-site mutation and metabolic mechanism in this region.

*An. gambiae* displayed large variations in resistance levels to carbamates and organophosphates. Although the wild populations were all resistant to bendiocarb, resistance was less marked to propoxur and fenitrothion, at WHO diagnostic concentrations. However, all these populations were very susceptible to pirimiphos-methyl. This resistance of the mosquito population to carbamate and fenitrothion would be due to the strong selective pressure that represents the use of insecticides in households for public health purposes, notably IRS using bendiocarb [21] and massive quantities of carbamates and organophosphates in agricultural settings in the department of Atacora. Indeed, in the cotton growing areas in Atacora, farmers use huge amounts of insecticides to avoid substantial yield reduction of their crops. Several studies showed that agricultural practices seem to have contributed to the emergence of insecticide resistance in Anopheles populations [8,12,32].

Cross-resistance to organophosphates and carbamates suggests the involvement of their common target-site: AChE-1 [33]. The high proportion of homoygous susceptible specimens, which survived the WHO bioassays, added to the low rate of ace-1R allele frequency suggest that the ace-1 mutation could not entirely explain *Anopheles gambiae* resistance to carbamate and organophosphate.

In the present study, the low ace-1 mutation frequency in sampled *An. gambiae* populations associated with the resistance to carbamate and organophosphate strongly supports the involvement of metabolic resistance based on the high activities of typical enzymes NSE, GST and MFO. Similar findings have been reported in *Culex quinquefasciatus* and *Anopheles gambiae* in four other sites in Benin , where greater oxidase and esterase activities were observed in *C.x quinquefasciatus* and *An. gambiae*, but where ace-1 was absent [34]. The same findings were also reported in the field experimental station of Pito (Cameroun) [35,36].

Considering the mutiple insecticide resistance mechanisms in *An. gambiae* s.l population from Atacora, resistance management becomes a particulary important issue. Insecticide rotation or synergistic use of different classes of insecticides to enhance the killing effects are being considered as alternative methods of vector control to slow down the spread of resistance genes.

## Conclusions

The present study revealed the simultaneous presence of multiple resistance mechanisms in malaria vector *An. gambiae* s.l population from Atacora. The co-implication of both metabolic and ace-1 resistance mechanisms in *An. gambiae* s.l may be a serious obstacle for the future success of malaria control operations based on LLINs and IRS. Altenative innovative vector control tools are therefore urgently needed to manage these complex mechanisms and to complement insecticide-based strategies.
